# The Seagrass Holobiont and Its Microbiome

**DOI:** 10.3390/microorganisms5040081

**Published:** 2017-12-15

**Authors:** Kelly Ugarelli, Seemanti Chakrabarti, Peeter Laas, Ulrich Stingl

**Affiliations:** Ft. Lauderdale Research and Education Center, Department of Microbiology and Cell Science, UF/IFAS, University of Florida, Davie, FL 33314, USA; kugarelli@ufl.edu (K.U.); seemanti@ufl.edu (S.C.); peeter.laas@ufl.edu (P.L.)

**Keywords:** seagrass, microbiome, sulfide, rhizosphere, holobiont

## Abstract

Seagrass meadows are ecologically and economically important components of many coastal areas worldwide. Ecosystem services provided by seagrasses include reducing the number of microbial pathogens in the water, providing food, shelter and nurseries for many species, and decreasing the impact of waves on the shorelines. A global assessment reported that 29% of the known areal extent of seagrasses has disappeared since seagrass areas were initially recorded in 1879. Several factors such as direct and indirect human activity contribute to the demise of seagrasses. One of the main reasons for seagrass die-offs all over the world is increased sulfide concentrations in the sediment that result from the activity of sulfate-reducing prokaryotes, which perform the last step of the anaerobic food chain in marine sediments and reduce sulfate to H_2_S. Recent seagrass die-offs, e.g., in the Florida and Biscayne Bays, were caused by an increase in pore-water sulfide concentrations in the sediment, which were the combined result of unfavorable environmental conditions and the activities of various groups of heterotrophic bacteria in the sulfate-rich water-column and sediment that are stimulated through increased nutrient concentrations. Under normal circumstances, seagrasses are able to withstand low levels of sulfide, probably partly due to microbial symbionts, which detoxify sulfide by oxidizing it to sulfur or sulfate. Novel studies are beginning to give greater insights into the interactions of microbes and seagrasses, not only in the sulfur cycle. Here, we review the literature on the basic ecology and biology of seagrasses and focus on studies describing their microbiome.

## 1. Seagrass Diversity and Ecology

Seagrasses are descendants of terrestrial plants that colonized the sea on at least three different occasions, starting around 100 million years ago [1,2]. They are aquatic angiosperms that form a coherent ecological group, but are quite diverse in terms of phylogeny [3] containing around 70 species of basal monocots that belong to four or five families (depending on the reference) in the order Alismatales. These families include *Hydrocharitaceae*, *Posidoniaceae*, *Zosteraceae*, *Cymodoceaceae*, and *Ruppiaceae*, with the two latter sometimes being considered as one family [1]. The morphology of seagrasses is very similar to other aquatic plants with the exception of the formation of filiform pollen (*Zosteraceae*, *Posidoniaceae*, *Cymodoceaceae*) and strings of spherical pollen (*Thalassia*, *Halophila*) [4,5,6].

### 1.1. Distribution

The total area covered by seagrass meadows is poorly known, but recent estimates range between 300,000 and 600,000 km^2^ [7,8]. All continents except Antarctica harbor seagrass meadows. The water depth to which seagrasses can grow is limited by the amount of light penetrating the water column of marine environments [9]. Depending on the species, seagrasses have been found to grow in a wide range of depths both subtidally and intertidally, ranging from 1 to 3 m to a maximum of 58 m [10]. The species diversity of seagrass meadows can vary from one dominant species in most of the coastal regions to as many as 14 in the tropics [11].

### 1.2. Adaptations to Marine Environments

Compared to terrestrial plants, seagrasses show adaptations to the marine environment and the concomitant high salinity. For example, Olsen et al. [1] demonstrates the absence of genes involved in stomatal differentiation and synthesis and sensing of volatile compounds in *Zostera marina*, the only genome-sequenced seagrass species so far, which allows its existence in the ocean. A reduction in the number of defense-related genes in *Z. marina* has been correlated to the absence of stomata that normally act as an entry point of pathogens and pests [1]. *Z. marina* has also lost most of its UV resistance genes while the number of light-harvesting complex B genes required for thriving in the low-light conditions of the sea increased [1,12]. To protect their cell walls against desiccation and strong osmotic pressure, seagrasses have regained the ability to produce sulfated polysaccharides, which promote water and ion retention in the cell wall, a feature lost in some terrestrial and freshwater plants [1]. 

Seagrass cell walls contain all of the typical polysaccharides of land plants, but also contain polyanionic, low-methylated pectins and sulfated galactans, similar to cell walls of most macroalgae. These cell wall constituents are important for ion balancing, nutrient uptake and O_2_/CO_2_ exchange through leaf epidermal cells [1]. Organic osmolytes as well as small cytoplasm:vacuole ratios also help maintain the osmotic equilibrium in marine environments [1,3].

### 1.3. Reproduction

Seagrasses reproduce both asexually and sexually. Their underground connection through a network of root-like structures called rhizomes intermittently give off shoots and lead to clonal growth and asexual reproduction. In sexual reproduction, male flowers produce pollen, which are distributed by the movement of the water or through small invertebrates [1,6,13,14]. A microsatellite study by Arnaud-Haround et al. [15] suggested that the sexual mating system of seagrasses is likely based on random large-scale dispersal, an important aspect for the ecology and evolution of seagrass. 

## 2. Seagrass Ecosystem Services

Seagrasses are mostly found in shallow marine waters, such as bays and lagoons, where they form vast meadows and fulfill important ecological services [16,17]. With a single acre producing over 10 tons of leaves per year, the dense, leafy canopies of seagrass beds are shelter, food and nursery for numerous animals, including commercially relevant fish and shellfish [18,19,20,21,22]. One acre of seagrass alone can support up to 40,000 fish and 50 million invertebrates [21]. Because of the rich biodiversity in seagrass beds, these meadows are being used as indicators to determine the health of coastal ecosystems [23,24,25,26]. Not only do seagrasses provide food and shelter, but they also buffer waves and trap fine sediments and particles suspended in the water column, thus increasing water clarity [3,27,28]. 

### 2.1. Seagrasses and Blue Carbon

Blue carbon is defined as the carbon acquired by oceans and coastal habitat [29]. Seagrasses play a major role in capturing and holding carbon and thereby act as a carbon sink [30]. They primarily capture carbon dioxide from the atmosphere through photosynthesis and subsequently buildup biomass. Various techniques have been used to determine the carbon pool in coastal ecosystems [31], and it has been estimated that the global seagrass community produces between 20.73 and 101.39 Tg C per year [25]. It was also reported that seagrass ecosystems accumulate as much as 19.9 Pg of organic carbon [32], which accounts for nearly 15% of the total global carbon storage [33,34]. As a byproduct of photosynthesis, one square meter of seagrass produces up to ten liters of oxygen per day, thereby constantly oxidizing and recycling key inorganic nutrients and increasing oxygen levels in the water column [35,36]. Consequently, the rise in oxygen levels in seagrass bed leads to a surge in macrophyte, algae and phytoplankton populations [35]. Howard et al. [37] discussed that while considering blue carbon storage, it is important to consider the stocks of both organic and inorganic carbon as they both might have an overall effect on the seagrass ecosystem. According to Lovelock et al. [38], 70–80% of the carbon stocks in the top sediment layer of the coastal wetlands has been lost as carbon dioxide emissions due to disturbance events over the past 40 years. The carbon dioxide emitted by destruction and disturbances of seagrasses as well as mangroves and marshes can cost between $6–42 billion annually [39].

### 2.2. Seagrasses as Food Source

Manatees, dugongs, green sea turtles and geese use living seagrass leaves directly as a food source, despite unfavorably high C:N ratios and high cellulose levels [18,36,40]. Manatees can consume 30–55 kg of seagrass daily [41,42], dugongs can eat between 28 and 40 kg, and sea turtles can eat up to 2 kg of seagrass per day [36]. Crustaceans and snails also occasionally consume seagrass leaves [36].

Many of the species that inhabit seagrass meadows tend to be carnivorous and feed on animals that live within the seagrass beds and other organisms that frequent the meadows [18,40]. Bacteria living epiphytically on the seagrass leaves provide a source of food for invertebrates such as crustaceans and snails, which in turn decrease the amount of debris in the seagrass meadows. Mucous released by these organisms can however coat seagrass leaves and obstruct sunlight [36]. Regularly, bacterial decomposition of dead seagrass plants provides detritus as food for worms, sea cucumbers, crabs, and filter feeders such as anemones and ascidians. Additionally, seagrass detritus and debris can be carried to the shore as well as to the deep sea, where they are available as food source for organisms farther away from the seagrass beds [36]. Further decomposition releases nutrients, such as nitrogen and phosphorus, which are reabsorbed by seagrasses and phytoplankton [40].

### 2.3. Seagrasses as Habitat and Nursery

Seagrasses serve as a keystone habitat for migrating coral reef species as well as thousands of other animals, including water birds and other frequent visitors. The sediments in seagrass meadows are mainly inhabited by invertebrate species consisting of echinoderms, crustaceans (including copepods, amphipods and ostracods), bivalves, polychaetes and nematodes in adult and immature stages [18,19,20]. Other animals living in the water above and within the seagrass beds include fish, shrimps, prawns, amphipods and isopods [18]. Seagrasses are also commonly used as nurseries by several fish including pollock, cod, herring and whiting [18,19,20].

### 2.4. Seagrasses Reduce the Number of Pathogens

In addition to providing shelter and food, seagrass ecosystems can reduce exposure to bacterial pathogens of fishes, invertebrates and humans. In the presence of seagrass meadows, a 50% reduction in the relative abundances of bacterial populations known to cause diseases both in humans and marine organisms, was observed [43]. An in vitro study by Kannan et al. [44] showed that phytochemicals isolated from seagrass tissues can kill or inhibit numerous bacterial pathogens. In general, seagrasses are effective at reducing the bacterial loads, which could benefit several organisms [44].

### 2.5. Seagrasses as Ecosystem Engineers

Seagrass meadows are important for the protection and stability of shorelines. Together with coral reefs and mangroves, seagrasses provide a barrier against the harsh waves commonly generated by storms and also help to prevent the loss of sediment [17]. Seagrass roots extend both vertically and horizontally, preventing the plant from being uprooted and providing shallow coastal areas with additional protection from erosion caused to strong wave and tide energy. Seagrasses can reduce wave and bed shear stresses nearshore by more than 60% [17]. A study by de los Santos et al. [45] examined the ability of seagrass species to withstand forces that mimic the strength of waves and currents. The study focused on the leaves of 22 species, concluding that temperate seagrasses are more capable of resisting breakage than tropical species because of constant exposure to harsher physical conditions [45]. Additionally, seagrasses play an important role in defining coastal areas, supplying biogenic detritus made of seagrass roots and debris, mollusks, echinoids, algae, foraminifera and bivalve fragments, all of which are species that reside within the meadows [46,47]. This deposited biogenic debris leads to calcareous, shelly beaches and sand flats or dunes commonly occurring in temperate regions such as southern and southwestern Australia [46,47]. In the Mediterranean Sea, meadows of *Posidonia oceanica* are the main carbonate source for the beaches and coastal systems, producing high levels of carbonate from its inhabitants [46].

When a seafloor area lacks seagrass communities, the sediments are more frequently stirred up by wind and waves [40]. A study by Moore [35] revealed that in the presence of ample amounts of seagrass during spring and early summer, the level of total suspended solids (TSS) in the water column was much lower than in unvegetated areas. During midsummer to fall, when seagrasses become less abundant, sediment and other materials resuspend and increase the levels of TSS [35]. As a result, water clarity decreases, affecting marine animal behavior and generally decreasing the recreational quality of coastal areas [40]. Seagrasses also trap and filter nutrients that come from land-based industrial discharge and stormwater runoff before these nutrients are washed out to sea where they can have detrimental effects on other sensitive habitats such as coral reefs [36,40]. Furthermore, seagrass meadows release parts of the nutrients they absorb from the sediment back into the water, incidentally supplying the surrounding environment with nutrients [36].

## 3. Seagrasses under Threat

Losses of seagrass habitats have been observed worldwide. Waycott et al. [28] estimated that nearly 29% of the global area covered by seagrasses has already disappeared since 1879, meaning that also the ecosystem services provided by seagrasses decreased by about one third. In Southeast Asia, a loss of up to 40% of the seagrass meadows has been reported [48,49,50], and in the Mediterranean about 30% has been lost [51]. The loss of seagrass beds could potentially cause large amounts of carbon dioxide emissions, contributing as much as 10% of the 0.5–2.7 Gt C per year released from changes in land use [32]. Several major die-offs have occurred in the past, for instance in the 1930s, 1980s and, more recently, in 2015 with turtle grass as the main species affected, although other species have also suffered [28,52,53,54,55,56]. Table 1 provides a list of different regions and the seagrass species affected.

### 3.1. Human Activity

The major cause for the loss of seagrass is human activity. Beach erosion and deposition of fine sediments partly disturb and alter seagrass beds. For instance, *P. oceanica* disappeared due to siltation caused by the building of embankments in Italy, while another seagrass species, *Cymodocea nodosa*, began to appear [105,106]. Not only does siltation alter seagrass meadows, but it also decreases seagrass health by blocking sunlight due to the increase of particles in the water column, as well as by altering sediment composition [64,105]. Human activities such as coastal development, damming, and waste disposal into the ocean also impact the health of seagrasses and hinder their ability to provide ecosystem services, which can contribute to global warming due to the loss of a carbon sink [18,107,108]. The rising temperatures of the ocean in turn distress seagrass meadows causing reduced shoot lengths, which lead to other complications such as lack of exposure to light [109].

One of the most destructive human activities is the eutrophication of coastal waters. Eutrophication is due to effluence of nutrients and coastal development, leading to increased nutrient concentrations in the sea that promote the growth of epiphytes such as algae, which negatively affect seagrasses by blocking sunlight [73,108,110,111,112,113]. The most common substances leaked into the ocean include nitrogen and phosphorous compounds [113], but other chemicals found in groundwater, such as radon and methane, also often seep into the ocean [114]. Other less destructive human activities include fishing and collecting mollusks and crustaceans [18,107,108].

### 3.2. Sulfide

Sulfide is highly toxic for animals and plants, and has been described as a major contributor to seagrass die-offs all over the world [3,59]. It is detrimental to several plant biological processes, such as photosynthesis, and is thought to be trapped in great amounts in the sediment of seagrass beds, where there is less iron available to sequester the sulfide [81]. Some species of seagrasses are more resistant to higher sulfide concentrations due to lesser biomass below the sediment, whereas turtle grass e.g., is less resistant [80]. Most marine and estuarine sediments are rich in sulfide due to activities of sulfate-reducing prokaryotes that perform the last step of the anaerobic food chain by oxidizing organic carbon and simultaneously reducing sulfate to H_2_S [115]. Several studies suggest that die-offs in the Florida and Biscayne Bays are caused by an increase in sulfide concentrations as a result of unfavorable environmental conditions and the activities of many different groups of heterotrophic bacteria in the sulfate-rich water-column and sediment [64,80]. For instance, a recent drought in 2015 lead to increased sulfide concentrations in the ocean, raising its toxicity and causing major losses of seagrass beds in the Florida Bay [55].

Low oxygen levels in the sediment lead to the uptake of toxic sulfide into the shoots [81,116,117]. Nonetheless, under normal conditions, seagrasses are able to withstand low levels of sulfide, probably partly due to microbial symbionts that detoxify sulfide by oxidizing it to sulfur or sulfate. However, an increase in sulfide concentrations has been shown to reduce shoot, rhizome and root production, eventually resulting in mass mortality of seagrasses [64].

### 3.3. Other Natural Threats 

Other natural causes of seagrass depletion include competition among species of seagrasses and seaweeds for habitat and resources [56,118,119], as well as diseases e.g., caused by the protozoan *Labyrinthula* [59,80,91,120,121]. Overgrazing by herbivores such as sea urchins can also contribute to the decaying seagrass population [122]. Furthermore, severe weather conditions, including hurricanes, can thin seagrass beds and deposit and erode sediments, thus altering the composition of both the water column and sediment which causes further deterioration of the seagrasses [123]. In general, factors that have a negative effect on seagrasses also negatively impact their microbial community, and damage the general health of seagrass meadows [124]. 

## 4. The Seagrass Holobiont

The concept of the holobiont [125], which emphasizes the importance and interactions of a microbial host with associated microorganisms and viruses and describes their functioning as a single biological unit, has been investigated and discussed for many model systems, although there is substantial criticism on a concept that defines diverse host-microbe symbioses as a single biological unit [126]. The holobiont and hologenome concepts have evolved since the original definition [127] and there is no doubt that symbiotic microorganisms are pivotal for the biology and ecology of the host by providing vitamins, energy and inorganic or organic nutrients, participating in defense mechanisms, or by driving the evolution of the host [128]. Although most work on host-microbe interactions has been focused on animal systems such as corals [129], sponges [130], or humans [131], there is a substantial body of literature on plant holobionts [132]. Plant-associated microbial communities impact both key components of the fitness of plants, growth and survival [133], and are shaped by nutrient availability and plant defense mechanisms [134]. Several habitats have been described to harbor plant-associated microbes, including the rhizoplane (surface of root tissue), the rhizosphere (periphery of the roots), the endosphere (inside plant tissue), and the phyllosphere (total above-ground surface area). In the following paragraphs, we review the existing literature on associations of seagrasses with microorganisms and discuss potential functional aspects of microorganisms that can impact growth and fitness of seagrasses.

## 5. Functions and Interactions of Seagrass Microbiome

**Figure 1:**
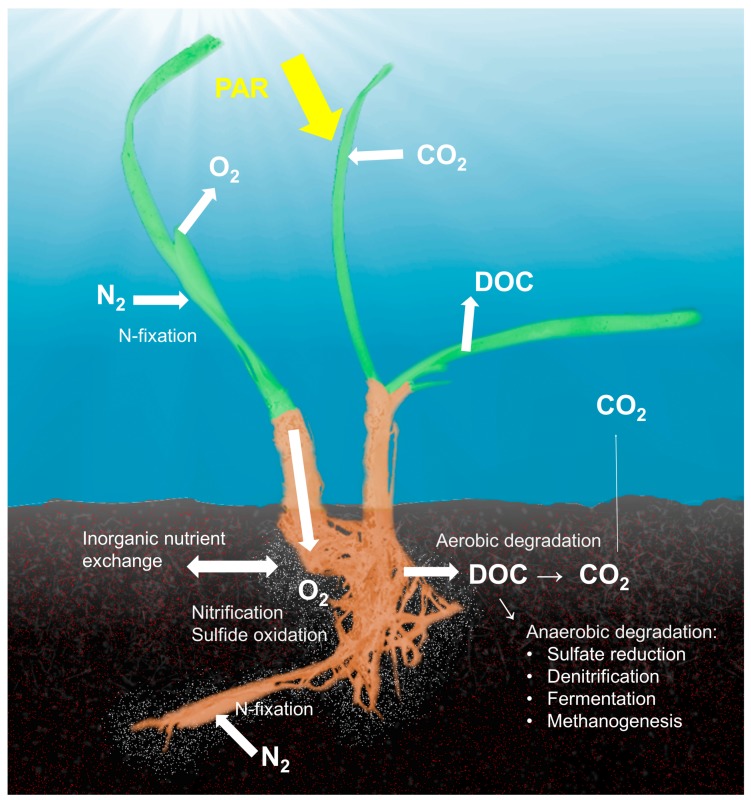
The most important interconnected processes within the seagrass holobiont are related to processes in the carbon-, nitrogen- and sulfur cycles. Photosynthetically active radiation (PAR) determines the photosynthetic activity of the seagrass plant that determines how much carbon dioxide is fixed, how much dissolved organic carbon (DOC) is exuded from the leaves and root system, and how much oxygen is transported into the rhizosphere. Oxygen transportation into the rhizosphere alters the redox conditions in the rhizosphere, differentiating it from the surrounding sediments that are usually anoxic and sulfidic.

Our knowledge of the seagrass holobiont has only recently started to take shape. Technological limitations and divergent sampling methods conducted in limited numbers of geographical areas and host species, along with a focus on specific members of the microbial communities instead of the total microbiome, lead to numerous individual results yet an incomplete understanding of the structure and function of the holobiont (Table 2). The literature on epiphytic algae and invertebrates has been summarized in detail elsewhere [135]; thus, this review focuses on the microbiome. The latest review on the seagrass rhizosphere communities was published more than ten years ago [136]. Metabolic capabilities and phylogenetic composition of microbial communities are unquestionably linked; however, most of the studies presented here have addressed either the structural or functional aspects of the microbiome. Earlier investigations have provided overwhelming evidence of direct links between the activities of seagrass hosts and their microbiomes (Figure 1). For clarity, these activities are discussed in relation to different biogeochemical cycles and protective functions.

### 5.1. Carbon Cycle

The physico-chemical parameters in the sediments are modified considerably by seagrasses. The diel photosynthetic activity of seagrasses is reflected in the amounts of dissolved organic carbon (DOC) and oxygen that are exuded through the roots and leaves [162,165,167,171]. Bacterial production rates can be two-fold higher during noon compared to nighttime in the seagrass rhizosphere, as opposed to nearby non-vegetated sediments [162,194]. At least for epiphytic bacteria on *Zostera marina* leaves, these exudates can account for most of the carbon needs [156]. In contrast, stable carbon isotope experiments carried out by Boschker et al. [47] in eutrophic ecosystems showed that seagrass-derived DOC in the sediments of *Zostera* spp. meadows only played a minor role for bacterial growth, and the authors suggested benthic algae as the main carbon source. However, subsequent studies carried out in oligotrophic seagrass beds found that seagrass-exuded DOC can be an important source of carbon for bacteria in the sediments [151,152,154]. Detrital plant material also adds carbon and other nutrients to the sediments of seagrass beds.

### 5.2. Nitrogen Cycle

Caffrey and Kemp [148] demonstrated that nitrification, denitrification and ammonification rates in the rhizosphere of *Z. marina* were significantly higher than in bare sediments, and that these processes were correspondingly influenced by the concentration of nitrate, inputs of organic nitrogen, and the release of O_2_ through plant roots. Ammonification is an important process catalyzed by the microbiome to cycle nitrogen species in seagrass systems, with the highest rates of ammonification having been recorded during the active growth season as seagrass biomass has a high seasonal turnover [148,168]. 

Nitrogen-fixing (diazotrophic) bacteria and archaea also play a very beneficial role for their host, especially in oligotrophic seagrass beds where nitrogen might be limiting. *N*-fixing bacteria are up to 300 times more abundant in the rhizosphere of *T. testudinum* and *Z. marina* than in bare sediments [164]. Diverse diazotrophic assemblages have been identified based on *nifH* gene sequences, which encode for the nitrogenase iron protein [137,138]. Sun et al. [192] could show that the copy numbers of *nifH* were significantly higher in eelgrass-vegetated meadows than in the surrounding sediments. Ammonia from nitrogen-fixing microorganisms in the rhizosphere can provide anywhere between a third to 100% of the nitrogen requirements of the plant [164,192,195,196,197]. Nitrogen fixation is not limited to the rhizosphere and high nitrogen fixation activities (up to 15.2 mg N m^−2^ d^−1^) have also been detected in the phyllosphere of *P. oceanica* in the Mediterranean Sea, potentially supplying the total nitrogen demand of the plant [137]. 

Similar to circadian patterns in bacterial production, diel cycles and seasonal changes in nitrogen fixation rates have been observed in sediments of seagrass beds [160]. Welsh et al. [196] and Agawin et al. [137] summarized many studies carried out in seagrass beds that demonstrate that *N*-fixation rates can be even two magnitudes higher in conditions with more light (highest rates in the summer and daytime). In Biscayne Bay, *N*-fixation rates of the *Z. marina* rhizosphere can be 20-fold higher in the summer compared to winter. Temperature was identified as an important factor for this change and it was shown that a decrease of 10 °C halved the rates [149]. Higher photosynthetic activity of the seagrass plant means that more oxygen is exuded to the rhizosphere [163,166], which has an impact on nitrogen fixation as nitrogenases can be irreversibly inhibited by dioxygen. Several mechanisms can be used by diazotrophs to overcome this, and Agawin et al. [137] suggested that *N*-fixing bacteria in the seagrass rhizosphere reside in anoxic microzones. Two-dimensional planar analyses of *Z. marina* root systems have shown that radial oxygen loss is not uniform and small areas in the tips of new roots are the main source of leakage, which can create suitable niches for anaerobic microbes along other parts of the roots [150,184].

### 5.3. Sulfur Cycle

Sediments in seagrass meadows are often anoxic and sulfate-reduction rates are many-fold higher in the rhizosphere than below the root zone or adjacent, non-vegetated sediments [153,158,198]. A recent publication summarized depth-integrated sulfate reduction rates reported by 16 previous studies carried out in seagrass beds [155]. Lactate- and acetate-utilizing sulfate-reducing bacteria are an order of magnitude more abundant in the *Halodule wrightii* rhizosphere than in nearby bare sediments [140]. Diel cycles have also been identified to impact sulfate reduction in the rhizosphere with rates nearly doubling during daytime [145,161,199,200]. In addition, sediments of *T. testudinum* meadows show strong seasonal patterns, as sulfate reduction rates can increase two-fold when seagrass is more actively growing [145,159,198,201]. Similar to nitrogen fixation, this process should be inhibited by the presence of oxygen, but root surface-associated sulfate reducers demonstrate a high tolerance towards oxygen [144]. Niches with reduced oxygen concentrations are formed by the activity of aerobic metabolism of microbes in the rhizosphere, but are also present in parts of the rhizosphere with lower oxygen leakage [150,184]. 

Sulfate reduction produces sulfide, a strong phytotoxin [202] that can lead to major seagrass die-off events [81,203]. Seagrasses are able to detoxify lower levels of sulfide through oxygenating their roots [204], integrating reduced sulfur into their biomass as thiols [205], and through the detoxifying activity of endosymbiotic, sulfide-oxidizing bacteria in lucinid bivalves, which colonize sediments of seagrass beds [2]. Sulfate-reducing bacteria are a dominant group involved in nitrogen fixation in the rhizosphere of *Zostera noltii* and other seagrasses [160,177,196], which could mean that for the seagrass holobiont, the presence of these bacteria is overall beneficial, especially when sulfide is neutralized by other members of the microbiome. Sulfur-oxidizing bacteria, which convert sulfide into non-toxic sulfate, are present in the rhizosphere and likely benefit seagrasses by detoxifying low levels of hydrogen sulfide. This hypothesis has been discussed by several recent papers that have observed a high abundance of presumably sulfide-oxidizing taxa within the rhizome-associated bacterial communities (e.g., [176,178,179]). Jensen et al. [184] suggested that the presence of the roots stimulates the growth of potential symbiotic sulfur-oxidizing taxa.

### 5.4. Protective Functions of the Seagrass Microbiome

Removal of phytotoxic compounds is an important function of the seagrass microbiome that benefits and increases the ecological fitness of the host. The symbiotic relationship between the seagrass plant and sulfur-oxidizers capable of detoxifying sulfide is discussed above. Similar mechanisms of bacterial detoxification of sulfide in the rhizosphere of rice have been known for 40 years [206]. Another phytotoxin, ethanol, is produced by the seagrass plant itself at night, when root tissues switch to fermentation due lack of oxygen [207] Cúcio and colleagues [176] suggested that sulfur-reducing members of *Desulfobacteraceae* can use ethanol as an electron donor and are able to detoxify it during night time. 

Seagrasses use a combined strategy of H_2_O_2_ production and caspase activity to combat fungal infections [208]. Cúcio et al. [176] suggested that some members of Actinobacteria in the seagrass rhizosphere might also provide protection against pathogens for the host. Some symbiotic bacterial epiphytes and especially endophytes of the seagrass *Enhalus acoroides* are natural antifoulants against harmful biofilm-forming bacteria [141]. 

While these studies hypothesize that microorganisms within the seagrass holobiont act as a defense mechanism, to the best of our knowledge this has not been convincingly proven to be a general feature of the seagrass microbiome. 

## 6. Structure of the Seagrass Microbiome

**Figure 2:**
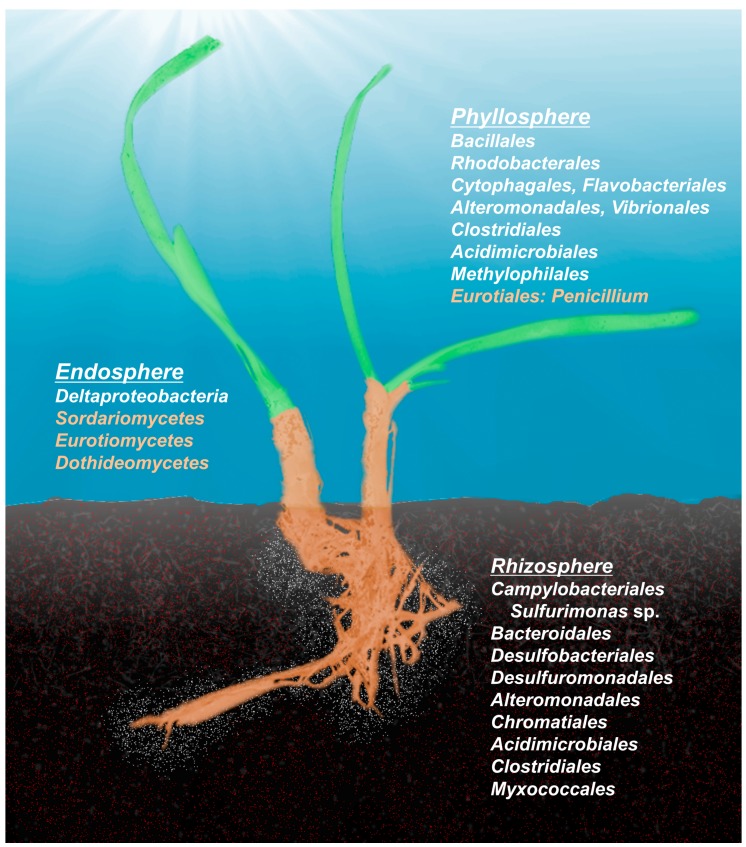
The most abundant bacterial (**white fonts**) and fungal groups (**orange fonts**) identified within different compartments of the seagrass holobiont.

The phylogenetic composition of microbial communities is interconnected with their functional capabilities. In order to understand the phylogenetic assembly of seagrass-associated microbial communities, the three major compartments of the microbiome (rhizosphere, endosphere and phyllosphere) have to be considered separately, because they represent different habitats with distinct interactions between the biotic and abiotic environments (Figure 2). In general, the phyllosphere offers niches that can be occupied by a larger variety of generalist taxa (like many aerobic organoheterotrophs), while the endosphere and rhizosphere provide niches to more specialized species (including organisms involved in transformations of different nitrogen and sulfur species). 

### 6.1. Phyllosphere

A recent global-scale study of the *Z. marina* microbiome revealed high variability and spatial turnover among leaf bacterial communities, which strongly resembled bacterioplankton communities of the adjacent coastal seawater [179]. Leaf-enriched taxa were dominated by members of the Betaproteobacteria, Planctomycetia, OM190, and Acidimicrobiia [179]. Similar results were obtained by investigating the leaf microbiome of *Z. marina* in coastal sites of the Baltic Sea, where the most abundant taxa matched key chemoorganotrophic bacterioplankton groups of the water, such as members of Alphaproteobacteria, Sphingobacteria, Flavobacteria and Verrucomicrobia [173,183,209,210]. 

Bengtsson et al. [173] demonstrated a co-occurrence of epibiotic eukaryotes and members of the bacterial microbiome on the leaves, with extensive variability in both groups correlated to water depth, leaf surface area and chlorophyll a content in the leaf biofilm. Previously, *Halophila stipulacea* leaves were shown to be colonized by bacteria associated with marine snow [193]. Similarly, taxa that also attach to particles and phytoplankton cells, including members of Bacteroidetes, were reported on leaves of *Z. marina* [186,211]. Together, these results indicate that the seagrass phyllosphere is mostly colonized by the surrounding heterotrophic bacterioplankton, and especially by groups that usually possess the ability to degrade polymers and are known to attach to surfaces and form biofilms (Figure 1). Marine fungi with similar metabolic capabilities are also frequent members of the phyllosphere of seagrasses [142,173,181,187,191]. At higher taxonomic ranks, the phyllosphere of *Z. marina* resembles the microbiomes of various marine algae [178]. Roth-Schulz and colleagues [212] concluded that seagrass phyllosphere communities are assembled mainly by taxa that specialize in surface-associated life-styles, but that in some cases certain host-specific features, such as the capability to degrade specific polysaccharides that are present in this environment, play an important role.

### 6.2. Rhizosphere

Overall, the bacterial abundances and biomass in the rhizosphere exceed those found in nearby non-vegetated sediments by about two-fold, but these numbers vary depending on the season [140,147,177,183,185,198]. In contrast to the phyllosphere microbiome, which resembles the surrounding bacterioplankton community, studies utilizing next-generation technologies have demonstrated that the seagrass rhizosphere microbiome exhibits significant differences in composition compared to adjacent vegetated sediment communities [124,176,178,184]. A global-scale study of the *Z. marina* microbiome revealed that the rhizosphere microbiome harbored a higher diversity and significant differences in composition compared to the phyllosphere [179]. However, the diversity of rhizosphere communities can be reduced by physical and chemical disturbances, including human activities [213]. Ettinger et al. [178] demonstrated significant shifts in microbial community composition within different areas of the rhizosphere itself, especially between the edge and the center of the rhizosphere. The study also revealed that sediment communities were much more diverse than rhizosphere communities, which indicates selective forces within the rhizosphere for certain taxa. One selecting factor is probably the labile organic matter that is being exuded from the roots, since certain heterotrophic organisms, such as members of the Bacteroidales and Myxococcales, are abundant in the seagrass rhizosphere [178,192]. 

Another important factor influencing the rhizosphere community composition is the generally anoxic condition of the sediments that are inhabited by seagrasses. Seagrass roots emit oxygen into the sediment [150,214] and thereby create a redox gradient in the rhizosphere, which forms specific niches for metabolically diverse bacteria. These redox gradients are essential for the presence and the activity of chemolithotrophic microorganisms. Consequently, seagrass rhizosphere microbiomes harbor a mix of aerobic and anaerobic members of the classes Alpha-, Gamma-, Delta- and Epsilonproteobacteria, Bacteroidetes and Clostridiales (Figure 1; [174,175,176,178,179,182,184,188,192]). Cúcio et al. [176] identified almost 3500 different taxa (97% 16 rRNA gene sequence similarity) from the rhizospheres of *Z. marina*, *Z. noltii*, and *Cymodocea nodosa* across the North-eastern Atlantic Ocean, out of which only 101 taxa were present in all samples (including replicates) and were considered as part of the core seagrass rhizobiome; 64% of these core taxa belong to Alpha-, Gamma-, Delta-, and Epsilonproteobacteria. The authors suggested that the main driver of the composition of seagrass rhizobiome communities is the host’s metabolism and that the resulting metabolic niches are not necessarily specific to a certain seagrass species. However, significant differences in the microbial community composition of a single seagrass species can be detected among different sampling sites (and their respective sediments), which indicates the influence of the surrounding sediments as a source for recruitment. Interestingly, many core taxa identified in this study were presumed to be active in the sulfur cycle and are likely directly involved in detoxification of sulfide or sulfur. 

### 6.3. Endosphere

Only limited information is available on endophytic microbes of seagrasses. Although previous experiments showed that only low activities of sulfate reducers can be detected in the cortex cells of *Z. marina* [145], Küsel et al. [140] demonstrated that sulfate reducers colonize 60% of the deepest cortex cells of *Halodule wrightii*. This study also reported the presence of acetogenic bacteria in the outermost cortex cell layers. Furthermore, the sulfate-reducing and *N*-fixing bacterium *Desulfovibrio zosterae* was isolated from surface-sterilized roots of *Z. marina* [189]. Additional studies identified organisms that were closely related to known endophytes: Cifuentes et al. [174] investigated sliced sections of rhizome material from the seagrass *Zostera noltii* and identified five species of Gammaproteobacteria closely related to *S*-oxidizing endosymbionts; Sakayaroj et al. [215] demonstrated that the seagrass *Enhalus acoroides* harbors a wide diversity of fungal endophytes, with 93% of them belonging to the classes Sordariomycetes, Eurotiomycetes and Dothideomycetes; two studies have isolated and identified fungi (members of Dothideomycetes and Leotiomycetes) from the rhizosphere of *P. oceanica* and determined that some isolates were closely related to endophytic species [181,191]. 

## 7. Conclusions about the Current Knowledge of Seagrass Microbiomes

Research over the past 40 years has shown elevated microbial activity within the seagrass holobiont compared to microbial communities occupying nearby non-vegetated environments. These activities include many important biogeochemical transformations within the nitrogen, sulfur and carbon cycles and are impacted by the physiology of the seagrass host. Some microbial activities, such as *N*-fixation in oligotrophic ecosystems or sulfide-oxidation in seabeds suffering from prolonged anoxia, can clearly improve the host’s fitness and its adaptation to certain habitats. The phylogenetic composition of the seagrass microbiome is influenced by microbial communities occupying the surrounding environment, but also significantly differs from them. The presence of a few reoccurring core taxa indicates specific niches provided by seagrasses. However, most ecological functions of the seagrass microbiome, including the core microbiome, and their interactions within the seagrass host are still unknown. No study so far has systematically addressed taxonomy and functional profile of the seagrass microbiome in parallel. Such studies would provide clues for key host-microbe interactions and further elucidate the multi-faceted roles of the microbiome in maintaining a healthy seagrass holobiont. These studies could improve our understanding of current and future seagrass die-off events, and possibly help provide strategies to mitigate or even avoid them.

## Figures and Tables

**Table 1:** List of seagrass species affected by die-off events in different regions of the world.

Region	Species Affected	References
Africa	*Cymodocea serrulata*, *Halodule wrightii*, *Halophila stipulacea*, *Thalassia hemprichii*, *Thalassodendron ciliatum*, *Zostera capensis*, *Zostera marina*	Nordlund et al., 2010 [57], de la Torre and Ronnback 2004 [58], Short and Green 2003 [59], Wakibya 1995 [60], Nasr and Aleem 1949 [61], Tackholm et al., 1941 [62], Gordon et al., 2008 [63]
Asia	*Cymodocea rotundata*, *Enhalus acoroides*, *Halodule pinifolia*, *Halodule uninervis*, *Halophila beccarii*, *Halophila decipiens*, *Halophila ovalis*, *Ruppia maritima*, *Thalassia hemprichi*, *Zostera asiatica*, *Zostera japonica*, *Zostera marina*	Unsworth et al., 2016 [48], Tomascik et al., 1997 [49], Nadiarti et al., 2012 [50], Short and Green 2003 [59], Halun et al., 2002 [64], Terrados et al., 1998 [65], Japar et al., 1995 [66], Japar et al., 1999 [67], Japar et al., 2001 [68], Mukai et al., 1999 [69], Parthasarathy et al., 1988 [70], Parthasarathy et al., 1991 [71], Short et al., 2011 [72]
Australia	*Amphibolis antarctica Halophila ovalis*, *Posidonia angustifolia*, *Posidonia australis*, *Posidonia sinuosa*, *Ruppia megacarpa*, *Zostera capricorni*, *Zostera muelleri*, *Zostera tasmanica*	Walker and McComb 1992 [73], Short and Green 2003 [59], Dennison and Abal 1999 [74], Larkum and West 1990 [75], Kirkman and Kendrick 1997 [76], Wells et al., 1991 [77], Silberstein et al., 1986 [78], Cambridge et al., 1986 [79], Short et al., 2011 [72]
North America	*Halodule wrightii*, *Halophila engelmannii*, *Ruppia maritima*, *Syringodium filiforme*, *Thalassia testudinum*, *Zostera marina*	Zieman et al., 1999 [53], Orth and Moore 1983 [52], Waycott et al., 2009 [28], Hall et al., 1999 [80], Borum et al., 2005 [81], Thayer et al., 1984 [82], Short et al., 1996 [83], Wyllie-Echeverria et al., 1999 [84], Lathrop et al., 2001 [85], Renn 1934 [86], Tutin 1938 [87], Sargent et al., 1995 [88], Hall et al., 1999 [80], Eleuterius 1973 [89], Sullivan 1979 [90], Short et al., 2002 [91], Hauxwell et al., 2001 [92]
South America	*Halodule wrightii*, *Ruppia maritima*	Short and Green 2003 [59], Phillips 1992 [93], Seeliger et al., 1997 [94], Oliveira et al., 1983 [95]
Europe	*Cymodocea nodosa*, *Posidonia oceanica*, *Zostera marina*, *Zostera noltii*	Short and Green 2003 [59], Blegvad 1935 [96], Helcom 1998 [97], Rasmussen 1973 [98], Baden et al., 2003 [99], Holmer and Bondgaard 2001 [100], Whelan and Cullinane 1987 [101], de Jonge and de Jonge 1992 [102], Frederiksen et al., 2004 [103], Komatsu et al., 1997 [104]

**Table 2:** Detailed overview on studies on the microbiome of seagrasses.

Structure, Function, or both (S, F, B)	Reference	Study Area	Methodology	Seagrass Species	Target	Compartment of Microbiome	Main Results
B	Agawin et al., 2016 [137]	Alcudia Bay, Mallorca, Spain	Sanger sequencing of *nifH* genes, acetylene reduction assay	*Posidonia oceanica*	*N*-fixing bacteria	Phyllosphere	Identified significant nitrogen fixation activity in phyllosphere.
B	Bagwell et al., 2002 [138]	Eastern Bahamas	DGGE to resolve PCR-amplified *nifH* sequences	*Thalassia testudinum*	*N*-fixing bacteria	Rhizosphere	Identified diverse diazotroph assemblages in the rhizosphere and found similarities to communities associated with an intertidal grass (*Spartina alterniflora*).
B	Kurilenko et al., 2010 [139]	Troitza Bay, Gulf of Peter the Great, Pacific Ocean	Cultivation	*Zostera marina*	*Granulosicoccus coccoides*	Phyllosphere	Described a new bacterial species.
B	Küsel et al., 1999 [140]	Santa Rosa Sound, Florida, USA	Phospholipid fatty acid analysis, cultivation, oligonucleotide probes	*Halodule wrightii*, *Thalassia testudinum*	Archaea, Bacteria	Endosphere, rhizosphere	Acetogenic bacteria were dominant on rhizoplane, and sulfate reducers in the endosphere.
B	Marhaeni et al., 2010 [141]	Central Java, Indonesia	Cultivation and antifouling test via agar diffusion method	*Enhalus* sp.	Bacteria	Endosphere, phyllosphere, rhizosphere	Identified epiphytic and endophytic bacteria that potentially act as natural antifoulants.
B	Newell et al., 1981 [142]	Chesapeake Bay, USA	Cultivation	*Zostera marina*	Fungi and Bacteria	Phyllosphere	Fungal biomass did not account for more than 0.5% of leaf mass; estimated bacterial productivity 1.4× standing stock per day.
B	Nielsen et al., 2001 [143]	Bay of Arcachon, France	Sulfate and acetylene reduction rate measurements, scanning electron microscopy, cultivation	*Zostera noltii*	Sulfate reducing and *N*-fixing microorganisms	Rhizosphere	Observed patchy distribution of bacteria on the roots and rhizomes. Addition of sucrose stimulated *N*-fixation and sulfate reduction significantly in the rhizosphere, but not in the bulk sediment.
F	Blaabjerg and Finster, 1998 [144]	Limfjorden, Denmark	Sulfate reduction rates using radiolabeled sulfate	*Zostera marina*	Sulfate reducers	Endosphere, rhizosphere	Demonstrated that root surface-associated sulfate reducers have high tolerance towards oxygen.
F	Blaabjerg et al., 1998 [145]	Limfjorden, Denmark	Sulfate reduction rates using radiolabeled sulfate	*Zostera marina*	Sulfate reducers	Rhizosphere	Sulfate reduction rates were 3 times higher in August than in April, and were significantly positively correlated to light intensity.
F	Boon et al., 1986 [146]	Moreton Bay, Australia	15N isotope technique	*Zostera capricorni*	Ammonium turnover, glycine utilization	Rhizosphere	Greater ammonification rates in rhizosphere than in adjacent bare sediments.
F	Boschker et al., 2000 [147]	Northwest Europe	Stable carbon-isotope ratios of bacteria, sediment organic matter and plants	*Zostera marina* and *Z. noltii*	Biogeochemistry of rhizosphere	Rhizosphere	Seagrass material was of limited importance as a bacterial carbon source.
F	Caffrey and Kemp 1990 [148]	Choptank River, USA	Rates of nitrification, denitrification and ammonification	*Zostera marina*	Nitrogen cycling	Rhizosphere	Microbial communities are responsible for key nitrogen transformations in the rhizospheres of *Z. marina* and *P. portfeliatus.*
F	Capone and Taylor 1980 [149]	Biscayne Bay, USA, and Bimini Harbor, Bahamas	Nitrogen fixation rates, acetylene reduction assay	*Thalassia testudinum*	*N*-fixing bacteria	Rhizosphere	Rates of *N*-fixation were 20-fold higher in the summer compared to winter. 10 °C drop in temperature halved *N*-fixation rates.
F	Frederiksen and Glud 2006 [150]	Svenstrup, Denmark	Planar O_2_ measurements	*Zostera marina*	Oxygen dynamics	Rhizosphere	Rhizospheres of seagrass are probably of minor importance for total benthic O_2_ uptake rates.
F	Holmer et al., 2001 [151]	Phuket Island, Thailand	Sulfate reduction rates; stable carbon isotope composition of seagrasses, sediments and bacteria	*Cymodocea rotundata*, *Thalassia hemprichii*	Carbon cycling, sulfate reducers	Rhizosphere	Determined that bacteria used organic matter derived from seagrasses and showed that the contribution of sulfate reduction to nutrient availability was low.
F	Holmer et al., 2004 [152]	Mallorca and Cabrera Islands, Spain	Stable carbon-isotope ratios of bacterial phospholipid derived fatty acids and sulfate reduction rates with 1-step distillation	*Posidonia oceanica*, *Cymodocea nodosa*	Carbon cycling	Rhizosphere	Seagrass detritus was a major bacterial carbon source, but its importance was decreased in areas with higher external nutrient loading.
F	Isaksen and Finster, 1996 [153]	Bay of Arcachon, France	Sulfate reduction rates using radiotracer method	*Zostera noltii*	Sulfate reducers	Rhizosphere	Rates of sulfate reduction were twice as high in rhizosphere as in equivalent layer of the unvegetated sediment.
F	Jones et al., 2003 [154]	Lower Laguna Madre, USA	Stable carbon isotope ratios in phospholipid fatty acids	*Thalassia testudinum*	Bacteria	Rhizosphere	Majority of sedimentary organic carbon originated from seagrass plants and provided important carbon source for bacteria.
F	Kim et al., 2017 [155]	Southern coast of Korea	Sulfate reduction rates and turnover of acid-volatile sulfur	*Halophila nipponica*, *Zostera marina*	Sulfur cycling	Rhizosphere	Sulfate reduction was more stimulated by the dissolved organic carbon exuded from the roots of *H. nipponica* compared to those at the roots of *Z. marina*.
F	Kirchman et al., 1984 [156]	Great Harbor, USA	Thymidine incorporation (bacterial production)	*Zostera marina*	Bacteria	Phyllosphere	Heterotrophic bacterial community of the phyllosphere is almost entirely supported by their seagrass host.
F	Koepfler et al., 1993 [157]	Laguna Madre and Baffin Bay, USA	Pore-water dissolved organic carbon concentrations	*Halodule wrightii*	Carbon cycling	Rhizosphere	In vegetated sediments, pore-water dissolved organic carbon concentrations were 25% higher and bacterial production rates were 4-times higher compared to unvegetated bare sediments
F	Lee and Dunton, 2000 [158]	Corpus Christi Bay and Laguna Madre, USA	Photosynthetically active radiation (PAR) collected with spherical quantum sensor and sulfide determined according to Cline (1969)	*Thalassia testudinum*	Biogeochemistry of rhizosphere	Rhizosphere	Determined diurnal dynamics of sulfide concentrations in pore water because of photosynthetically produced oxygen being transported to below-ground seagrass tissues.
F	López et al., 1995 [159]	Mediterranean Sea, Spain	Ammonification rates; *exoproteolytic* and *exoglucosidase* activity	*Posidonia oceanica*	Bacteria	Rhizosphere	Benthic bacterial activity is directly related to seagrass productivity.
F	McGlathery et al., 1998 [160]	Limfjord, Denmark	Perfusion technique (acetylene reduction)	*Zostera marina*	*N*-fixing bacteria	Rhizosphere	Nitrogen fixation activity was about 3-times higher in vegetated than unvegetated sediments.
F	Moriarty et al., 1985 [161]	Pelican Banks, Australia	Thymidine incorporation and phospholipid method for bacterial production; Sulfate reduction and methane production rates	*Zostera capricorni*	Bacteria	Rhizosphere	Spatial variability overwhelmed seasonal variability in case of seagrass productivity and bacterial productivity; strictly anaerobic bacteria.
F	Moriarty et al., 1986 [162]	Northern Gulf of Mexico, USA	Stable isotope labeling experiments	*Halodule wrightii*	Carbon cycling	Phyllosphere, rhizosphere	On average 11% of the fixed carbon was exuded into the sediments and 1% into the water column. It took 6 h for the fixed carbon to be translocated from leaves to roots.
F	Oremland and Taylor 1977 [163]	Caesar Creek, USA, and Bimini Harbor, Bahamas	Chemical analyses of gas bubbles	*Thalassia testudinum*	Biogeochemistry of rhizosphere	Rhizosphere	Observed O_2_ transport via rhizomes to sediments and diurnal fluctuation of this process due photosynthetic activity of the seagrasses.
F	Patriquin and Knowles 1972 [164]	Clam Cove, Dear Island, Canada	Cultivation and acetylene reduction assay	*Thalassia testudinum*, *Zostera marina*	*N*-fixing bacteria	Rhizosphere	Estimate that *N*-fixing microbes in the rhizosphere can fully support nitrogen requirements of the seagrass host and that their abundance is 50–300 times higher than in unvegetated sediments.
F	Penhale and Smith 1977 [165]	Newport River estuary	Release of DOC using radiocarbon techniques	*Zostera marina*	Carbon cycling	Phyllosphere, rhizosphere	Excretion rates in the dark were much lower than in the light.
F	Sand-Jensen et al., 2005 [166]	Roskilde Fjord, Denmark	O_2_ microelectrodes	*Zostera marina*	Oxygen dynamics	Rhizosphere	Determined diurnal dynamics of oxygen concentrations in the rhizosphere.
F	Smith et al., 1984a [167]	Back and Bogue Sounds, USA	Total net ammonification by rhizoplane microflora, cultivation	*Zostera marina*, *Halodule wrightii*	Rhizoplane bacteria	Rhizosphere	Rates of ammonification were inversely related to the active growth season of these seagrasses.
F	Smith et al., 1984b [168]	Great Harbor at Woods Hole, USA	Polarographic two-chambered apparatus fitted with O_2_ electrodes	*Zostera marina*	Oxygen dynamics	Rhizosphere	O_2_ transport to the root-rhizome system supported aerobic root respiration.
F	Törnblom and Søndergaard, 1999 [169]	Roskilde Fjord, Denmark	Leucine and thymidine incorporation (bacterial production)	*Zostera marina*	Bacteria	Phyllosphere	Observed a strong correlation between bacterial production (leaf attached communities) and host’s primary production. Biomass production of microbiome was very high compared to the standing stock of bacteria.
F	Welsh et al., 1996 [170]	Bassin d’Arcachon, France	Acetylene reduction and cultivation	*Zostera noltii*	*N*-fixing bacteria	Rhizosphere	Acetylene reduction rates up to 4-fold greater in the light compared with those measured in the dark. Sulfate-reducing bacteria were the dominant component of the nitrogen-fixing microflora.
F	Wetzel and Penhale 1979 [171]	Puget Sound and northern Gulf of Mexico, USA; Bimini Harbor, Bahamas	Stable isotope tracers	*Halodule wrightii*, *Thalassia testudinum*, *Zostera marina*	Transport and excretion of carbon by seagrasses and their epiphytes	Phyllosphere, rhizosphere	Large portion of the inorganic carbon taken up by the rooting tissue was transported through the leaves and released into the water.
F	Williams et al., 2009 [172]	Florida Bay, USA	Stable carbon isotope ratios in phospholipid fatty acids	*Thalassia testudinum*	Pelagic, epiphytic, and sediment surface bacteria	Phyllosphere	Bacterial communities consistently incorporated seagrass-derived organic matter (13–67% of bacterial-specific stable carbon isotopic signatures).
S	Bengtsson et al., 2017 [173]	Baltic Sea, Germany	Amplicon sequencing of 16S and 18S rRNA genes	*Zostera marina*	Bacteria and Eukaryotes	Phyllosphere	Observed local variation of microbiomes and found correlation between prokaryotic microbiome and eukaryotic epibiont communities.
S	Bourque et al., 2015 [124]	Biscayne Bay, USA	T-RFLP	*Thalassia testudinum*	Bacteria	Rhizosphere	Evaluated two restoration methods and found that undisturbed reference seagrass sediments had more complex microbial communities than disturbed and restoration sites.
S	Cifuentes et al., 2000 [174]	Bassin d’Arcachon, France	Sanger sequencing of 16S rRNA genes, clone libraries	*Zostera noltii*	Bacteria and Archaea	Endosphere, rhizosphere	Report sulfate-reducing bacteria and *S*-oxidizing endosymbionts, as well as members of Crenarchaeota and Euryarchaeota.
S	Crump and Koch 2008 [175]	Chesapeake Bay, USA	PCR-DGGE and Sanger sequencing of clone libraries	*Zostera marina*	Bacteria	Phyllosphere, rhizosphere	Leaves were dominated by typical marine Alphaproteobacteria, while roots hosted a diverse microbial assemblage.
S	Cúcio et al., 2016 [176]	North-eastern Atlantic Ocean	Amplicon sequencing of 16S rRNA genes	*Zostera marina*, *Z. noltii*, *Cymodocea nodosa*	Bacteria	Rhizosphere	Rhizobiomes were similar in one geographic region, but were significantly different from the sediment bacterial communities.
S	Donnelly and Herbert 1999 [177]	Bassin d’Arcachon, France	Cultivation; light and scanning electron microscopy	*Zostera noltii*	Bacteria	Rhizosphere	Sulfate reducing bacteria were identified as the key group of bacteria involved in *N*-fixation in the rhizosphere.
S	Ettinger et al., 2017 [178]	Bodega Bay, USA	Amplicon sequencing of 16S rRNA genes	*Zostera marina*	Bacteria	Phyllosphere, rhizosphere	Taxa that differ significantly between sample types and sites are closely related to ones commonly associated with various aspects of sulfur and nitrogen metabolism.
S	Fahimipour et al., 2017 [179]	Multiple locations across the Northern Hemisphere	Amplicon sequencing of 16S rRNA genes	*Zostera marina*	Bacteria	Phyllosphere, rhizosphere	Provides evidence for a core eelgrass root microbiome (*Sulfurimonas* the most dominant taxon).
S	Glazebrook et al., 1996 [180]	Pelican Banks, Australia	Fluorescent microscopy studies with labeled antibodies	*Zostera capricorni*	*Alteromonas* sp.	Rhizosphere	Members of *AIteromonas* sp. were more abundant in rhizosphere than in bare sediment.
S	Gnavi et al., 2014 [181]	21 isolates obtained by Panno et al., 2013	Sanger sequencing of ITS and 28S rRNA genes	*Posidonia oceanica*	Fungi	Phyllosphere, rhizosphere	Identified several putative new species belonging to orders *Pleosporales*, *Capnodiales* and *Helotiales*.
S	Green-García and Engel 2012 [182]	Cedar Key, USA	Sanger sequencing of 16S rRNA genes, clone libraries	*Thalassia testudinum*	Bacteria	Rhizosphere	41% of the clones were more closely related to each other than to sequences retrieved from the other habitats.
S	Jankowska et al., 2015 [183]	Southern Baltic Sea, Poland	Epifluorescence microscopy	*Zostera marina*	Bacteria	Rhizosphere	Reported significantly higher bacterial cell numbers and bacteria biomass in rhizosphere compared to bare sediments.
S	Jensen et al., 2007 [184]	Roskilde Fjord, Denmark	T-RFLP and Sanger sequencing of clone libraries	*Zostera marina*	Bacteria	Rhizosphere	Bacterial community associated with the roots of *Z. marina* differs from the bacterial community in the bulk sediment.
S	Jiang et al., 2015 [185]	Xincun Bay, China	Cultivation and Sanger sequencing of 16S rRNA genes	*Cymodocea rotundata*, *Enhalus acoroides*, *Thalassia hemperichii*	Bacteria	Phyllosphere	Diversity of the bacterial communities in the sediment was higher than that associated with seagrass.
S	Kurilenko et al., 2007 [186]	Troitza Bay, Gulf of Peter the Great, Pacific Ocean	Cultivation based experiments	*Zostera marina*	Bacteria	Phyllosphere	Demonstrated symbiotrophic relationships between seagrass and epiphytic bacteria (selective adhesion).
S	Ling et al., 2015 [187]	Xincun Bay, China	PCR-DGGE, quantitative PCR and Sanger sequencing of clone libraries	*Enhalus acoroides*	Fungi	Rhizosphere	Fungal community of rhizosphere changed significantly in response to polycyclic aromatic hydrocarbons.
S	Mejia et al., 2016 [188]	Gulf of Aqaba, Israel	Amplicon sequencing of 16S rRNA genes	*Halophila stipulacea*	Bacteria	Phyllosphere, rhizosphere	Proposed assessing the ecological status of seagrasses using their microbiome.
S	Nielsen et al., 1999 [189]	Lørgstrar Broad, Denmark	Cultivation	*Zostera marina*	Sulfate reducers	Rhizosphere	Isolated a novel species of the genus *Desulfovibrio.*
S	Novak 1984 [190]	Gulf of Naples, Italy	Scanning electron microscopy	*Posidonia oceanica*	Epiphytic microorganisms	Phyllosphere	Observed dynamically changing system of interactions between the host plant, environmental and epiphytic community (also within).
S	Panno et al., 2013 [191]	Riva Trigoso Bay, Italy	Cultivation, microscopy, Sanger sequencing of ITS	*Posidonia oceanica*	Fungi	Phyllosphere, rhizosphere	Mycoflora associated to *P. oceanica* is very rich and characterized by fungi that are able to degrade and detoxify lignocellulose residues in presence of high salt concentrations.
S	Sun et al., 2015 [192]	Swan Lake, Rongcheng Bay, China	Amplicon sequencing of 16S rRNA genes	*Zostera marina*	Bacteria	Rhizosphere	Metabolically versatile and oxygen-tolerant anaerobic bacterial taxa were enriched in vegetated sediments.
S	Weidner et al., 1996 [193]	Gulf of Aqaba, Israel	ARDRA (amplified rDNA restriction analysis)	*Halophila stipulacea*	Bacteria	Phyllosphere	Improvement of the ARDRA method.
